# "The Hospital" Nursing Mirror

**Published:** 1897-07-24

**Authors:** 


*The Hospital*, July 24, 1897.

### "The Hospital" Nursing Mirror.

Being the Nursing Section of "The Hospital."

[Contributions for this Section of "The Hospital" should be addressed to the Editor, The Hospital, 28 & 29, Southampton Street, Strand, London, W.C., and should have the word "Nursing" plainly written in left-hand top corner of the envelope.]

### News from the Nursing World.

### PRINCESS CHRISTIAN AT NORWOOD.

On the 16th inst. the Princess Christian visited Norwood Cottage Hospital, visiting a ward named after the Empress Frederic, and inspecting the building, after which she was entertained by Mr. and Mrs. Tritton at Bloomfield, where she kindly consented to receive some purses in aid of the institution. A sum of £3,000 has been collected in honour of her Majesty's reign, which is to be devoted to the support of the "Hokkaido" bed at the hospital, and one at the Convalescent Home, Bexhill. The route was decorated, and the Princess was loyally welcomed.

### THE QUEEN'S NURSES FUND.

The Duke of Westminster's Committee hope to realize £100,000 for the Queen's nurses before closing this fund. £68,000 has been raised.

### NURSING STAFF FOR CANCER PATIENTS.

The new cancer wing at Middlesex Hospital, of which the Princess Christian laid the foundation-stone last Thursday, will accommodate 36 patients, 11 nurses, and a superintendent sister or matron. The matron's rooms will be on the ground floor, as will be the nurses' dining room. Each nurse will be provided with a separate bed-room on the second floor, whilst the kitchens will be over the central offices on the third floor.

### JUBILEE FESTIVITIES AT THE GREAT NORTHERN CENTRAL HOSPITAL.

Those excellent friends of the hospital, the Ladies' Association, did not allow the opportunity afforded by the Queen's Diamond Jubilee to slip, without marking the occasion in their own kindly fashion by entertaining the patients and nursing staff. Early in the afternoon of Friday, the 2nd inst., festivities began with music and singing provided in each of the wards by Mr. and Mrs. Ford and friends. This was followed by a sumptuous tea, of which nearly all were able to partake with appetite and enjoyment, and each patient was afterwards presented with a Jubilee cup of elegant design as a memento of the occasion. In the evening, Mr. C. T. Murdoch, M.P. (the chairman of the Hospital Committee), and Mrs. Murdoch (vice-president of the Ladies' Association) received the nursing staff in the board-room, and distributed amongst the sisters and nurses a number of charming gifts of silver-plated tea-table requisites, Doulton ware cups, and other presents. A large company of friends of the hospital were present, and partook of tea, and strawberries and cream; while Mr. and Mrs. Ford discoursed delightful music and song for their entertainment.

### VETERAN NURSES.

Her Majesty summoned to Windsor four nurses—Sisters Mary Ellen Ellis, Mary Stanislaus Jones, Mary Anastasia Kelly, and Mary de Chantal Huddon—who served under Florence Nightingale during the Crimean War, in order to bestow upon them the decoration of the "Red Cross." A Royal carriage met them at the station on July 9th, and luncheon was provided at the Castle, after which the Queen received them, and herself gave to each the decoration. It is like a whiff of a pot-pourri jar, this gracious act of remembrance. These ladies, the youngest of whom is now 69, and the oldest 81, left their Bermondsey convent in 1854, and faced privation and hardship in the dark days of the past, to carry help to our wounded soldiers in the East. Their task done, Cardinal Wiseman claimed their services for the new Catholic hospital which he opened in Great Ormonde Street; and now, in the day of rejoicing, their Sovereign calls them from their seclusion to receive an honour of which they are worthy.

### THE NIGHTINGALE FUND.

Once more we have the record of a year's good work done by the Fund given by the Nation to Florence Nightingale, and by her to the service of the Nation, before us. Out of an income of £2,229, £167 is carried on to next year's credit. Out of 39 probationer-nurses who have completed their year's training, 34 have been taken on to the staff at St. Thomas's Hospital. Out of the 1,375 candidates entering the school since its foundation, 825 have passed on to some approved school; and last year 23 nurses trained in the schools have been appointed to more or less important positions, there being six matrons amongst the number. Miss Florence Haig-Brown, who accepted the post of "Home Sister," in place of Miss Crossland, resigned, carries on instruction of candidates on the same lines as her predecessor, and the usual courses of lectures are given by medical men. During the year one nurse left to work at plague-stricken Bombay, and one joined the Army Nursing Service. Fourteen former probationers, having completed their term of service as parish infirmary nurses, have received higher posts on the ladder of success.

### FESTIVITY AT OSWESTRY.

The Record Reign was celebrated in right royal style at the Cottage Hospital, and fortunately all the inmates but one were able to participate in the rejoicings. The buildings and grounds were profusely decorated, and illuminated at night. Nurses, servants, and patients wore a commemorative medal, the gift of the vicar, the matron herself fastening them in place. Caeglas was thrown open for two days, and an abundance of dainties, a large number of visitors, and plenty of band music, with a fancy dress cycle parade, provided a most enjoyable treat, for which the matron, Miss Scott, received hearty thanks.

### THE IRISH BISHOPS AND PAUPER NURSING.

Individually, no sane-thinking creature can approve of pauper nursing. A pauper is one who has failed in life, generally—we had well-nigh said always—from some mental or moral deficiency; and that the sick and old should not be abandoned to the mercy of such is self-evident. But it is a difficult thing to get individuals to act collectively, and, therefore, we are heartily glad that the Irish Roman Catholic Episcopacy has sent the following reply to Mr. Thomas Kennedy hon. secretary of the Irish Workhouse Association, in reply to his letter asking counsel in this matter: "I am directed to state, for the information of your executive committee, (*a*) that the prelates unanimously condemn the system of employing pauper inmates for the nursing of the sick in the workhouse wards as it is carried on at present; (*b*) that they cordially endorse the recommendation of engaging the services of skilled or trained nurses to attend upon the workhouse sick during the hours of the night."

### STATE HELP TO TRAIN NURSES.

In France the State recognises the importance of gratuitous medical help to the poor, and the General Council of the Department of Hérault has recently voted a tax of half a centime for this purpose. The council is also desirous of seeing professional schools for nurses established in all towns where there are the requisite educational facilities. In the event of such schools being founded only nurses holding good diplomas will be eligible for appointments.

### LADY ABERDEEN AND THE CANADIAN NURSES.

Lady Aberdeen is working hard to make the new order of nurses which she is desirous of establishing a success. It is to be called the Victorian Order, and candidates for work in its ranks must pass a suitable examination by a committee of the medical men of the Dominion. The idea is to provide nurses for scattered districts, and to form the nucleus of cottage hospitals when the need arises. We hear that the scheme promises to be successful.

### NURSING AT THE CITY OF LONDON INFIRMARY.

We are very glad to hear on excellent authority that in spite of the antiquated system of nursing which prevails at the City of London Infirmary, the institution has never enjoyed better service in this respect, nor possessed a more efficient staff, or one animated by a more loyal and excellent spirit than the present one.

### A NURSING HOME.

Miss Florence Burrell gave an "At home" at 59, Weymouth Street, W., on the 30th ult. She has taken up the nursing home founded by the late Miss Carty, to whom she was secretary. There are 30 nurses on the staff, all of whom have three years' training, and hold certificates. The principle on which the home is conducted is co-operative.

### MEDICAL MEN AND DISTRICT NURSING.

Good not infrequently comes out of evil. It is unpleasant to have to record the occurrence of friction between medical men and district nursing associations; yet, unless the rules of such association are carefully drawn, and a certain willingness exists on both sides to co-operate for the general good, differences are sure occasionally to arise, and in seeking for the good which may have come out of the unfortunate differences at Honiton, to which we alluded last week, we find it in the very definite statement in the new rules of the Honiton District Nursing Association in regard to the relation between the patient and the nurse. There can be no doubt that what the doctors dislike, not at Honiton alone, but everywhere, is "interference" between them and their patients. Sometimes this sort of objection is carried to an absurd extreme, and medical men are to be found who lump together nurses, district visitors, parsons, and charitable people of all sorts as busybodies, to be kept at arm's length as much as may be. All this, however, arises from a feeling which, in its root, is good, namely, that the doctor is responsible for the progress of his patients; and we can quite admit that it is reasonable that a doctor should object to a nurse being intruded by any third person between himself and his patient. The Honiton rules, however, lay it down definitely that the application for a nurse must be made in writing, or on a printed form signed by the applicant, *i.e.*, the patient or the person in charge. The association provides the nurse, but only at the request of the patient, and under such circumstances, if the doctor objects, his quarrel lies with his patient and not with the association. But if the nurses are judicious, and the ladies are wise, the advantages of district nursing are so great that we feel sure that no quarrel is likely to be maintained for long.

### ASHTON-ON-MERSEY NURSES' HOME.

Lady Brooks laid the foundation-stone of the nurses' home, which is to cost about £2,000. Sir William Cunliffe Brooks gave the site, and the greater part of the sum required is promised.

### THE BISHOP OF EXETER'S GARDEN PARTY.

The Bishop of Exeter and Mrs. Bickersteth entertained 290 guests from the nursing staffs of the various institutions throughout the county of Devonshire at a garden party. Lunch was served at one p.m. in the grounds, and the post office band provided music. This is the first of a series of entertainments, and only a portion of nurses were present.

### SHORT ITEMS.

The Grant Road Hospital staff, Bombay, gave Mother St. Agnes and her colleagues an ovation on the occasion of their farewell. Dr. McCabe Dallas gave each nurse a medal in token of the esteem and gratitude in which the Catholic Sister and their work were held.—The nursing staff at Coombe Hospital, Dublin, has a character for being efficient and popular, and probationers, when their term of training is over, attain excellent positions. Dr. Kidd, in moving the adoption of the year's report, said that the accommodation for the staff was insufficient, and he thought the opportunity a favourable one to ask the public for increased support.—A site has been obtained for the proposed Barry Nursing Home. It is situated on the north side of Woodland Road, near Court Road, Barry Docks, and is one-third of an acre in extent. The lease is for 999 years, at £9 per annum. The one drawback is that it is some distance from the station, but it is in the centre of a rising neighbourhood. About £600 has been promised towards the erection of the building, and about £200 more will be needed.—Miss K. Twining, founder and superintendent of the Plaistow Maternity Charity, was unable to be present at the recent meeting of the institution, held at Stafford House, on account of illness.—The scheme to provide Festiniog with two trained nurses is being actively pushed by Mrs. Holland Caerdeon, and several handsome subscriptions have already been promised.—The Guardians of Halifax Union have, after some discussion, consented to a greater variety in the dietary of the nurses. The cost will probably not be greater.

### Practical Aspects of a Nurse's Life.

By Sister Grace.

### III.—PROBATIONERS IN MEDICAL WARDS.

### Patience Required.

Patience is a necessary virtue in all nursing, but it is, I think, more required in medical than in surgical work. Medical patients are much more peevish, fretful, and exacting than surgical, because, to use their own expressive term, they are "more ill in themselves." Those suffering from various forms of heart disease and those who are dropsical are extremely restless, constantly demanding change of position, wanting to get in and out of bed, to be propped up, to be laid down, in the vain hope that the terrible feeling of oppression they experience may be lessened. Each new position being found impracticable, the same process begins again, and so it goes on from day to day. It requires the exercise of enormous patience and gentleness to bear with this distressing state of things, and great experience to know to what extent it is advisable or safe to humour the patient. Whilst you are a probationer you will receive distinct orders on the subject, but when compelled to refuse the persistent and fretful requests of the patient, do it pleasantly and gently, and never let them see that your patience has worn thin.

Many a nurse, by no means clever or gifted, surpasses all others in her power of giving comfort and solace to the suffering from the sweet unselfishness that has prevented her from ever showing weariness or impatience under the most trying circumstances. Let me here remind you that you must not expect much display of gratitude from your patients; it is not the right spirit in which to begin your work, and will be most assuredly disappointed; in novels we read of many instances of life-long gratitude (most effectively expressed) to the hospital nurse, but in real experience we do not, I think, often meet with it. Patients take all their nurse's ministrations as a matter of course and as only their due. I remember one old man boldly announcing his conviction of our walk in life to a fellow patient, "Them gals is well paid to wait upon me, and I mean to take it out on 'em." That old gentleman, who came from the workhouse, and was therefore unusually hard to please, had the courage of his opinions.

You must remember that illness and suffering tend towards selfishness. The sick man is constantly thinking about himself, and imagines that the whole world revolves round his bed. The less you expect gratitude the more probably you will meet with it, for it will argue that you are not thinking of yourself. Remember Who it was Who said, "Inasmuch as ye have done it unto the least of these, ye have done it unto Me," and you will have your reward.

### Rheumatism.

Rheumatism in its various stages is one of the diseases met with in the medical wards, and from the suffering it involves gives great scope for the exercise of careful, tender, and skilful attendance. The patient perspires very much, and the perspiration has a peculiar sour odour impossible to mistake after having once made acquaintance with it. It is necessary that he should be laid between blankets and wear flannel night-shirts. In rheumatic fever the suffering is so acute the weight of the bed clothes must be supported by a cage. Frequent washing of the patient in hot water and continual changing of the blankets is necessary, and as the slightest movement gives acute pain it is not an easy matter to accomplish; but by this time you will have acquired some skill (we will hope) in the management of helpless patients.

### Hot Air Baths.

You will probably often be called upon to help the sister in the administration of hot air baths, which in some forms of kidney disease, &c., afford signal relief. Note carefully all she says, as it is quite possible to burn a patient seriously if due precautions are not used. All hospital appliances for this purpose differ a little, but the principle is the same. The patient must be laid between blankets without his shirt, the mattress being protected from the absorption of perspiration by a macintosh placed under the lower blanket, then a cylindrical cage of wicker work is put over the patient, and so gives free access to the hot air which is introduced at the foot of the bed by means of a long bent chimney coming from the lamp. The doctor or sister will carefully watch the patient's condition during the progress of the bath, and when it is over the patient must be rapidly dried and rubbed with hot towels, and put into a fresh hot woollen shirt and clean, hot blankets. Your common sense will tell you how serious and fatal a thing a chill would be to a patient after this treatment. You will probably be told to give the patient nourishment in some form when he is again settled down to rest.

### Delirium.

You will not be long in the wards before you encounter delirium in one of its many forms. Those suffering from pneumonia and typhoid fever are very subject to it, especially if they have been hard or continual drinkers. The treatment of delirious patients can only be learnt by experience. Some patients are violent, some are only constantly restless. Much tact is required in controlling them without unnecessary thwarting of every movement. I think the difficulties are increased when the patient is suffering from some injury or operation, because movement then is more detrimental. Having been for some years sister of male accident and operation wards I have had considerable experience of delirium tremens. Men who have been hard drinkers and are suddenly struck down by an accident or from the need of some operation are prostrated in the midst of their usual work and course of free living are sadly often attacked by alcoholic delirium. With operative work these cases frequently prove fatal, for what chance has a man, who has, perhaps, undergone some form of abdominal operation, and who tosses in perpetual delirium? It is important not to show any fear whatever your real feelings may be; you will lose all control over your patient if he is aware of your timidity. Nurses are not so severely tried in these respects as they were even ten years ago; the authorities are beginning to realise the danger and impropriety of leaving nurses to contend alone with dangerous lunatics, but even with the improved state of things which exist at present you will probably be often placed in situations that try your courage and call for the exercise of that peculiarly British quality that we call "pluck." You must not imagine that all delirious patients have been drinkers, that would be a great error; there are many other causes, as you will learn as you make progress in your profession.

### Appointments.

### MATRONS.

Southport Infirmary.—Miss Alice Clark was appointed on July 14th Matron of the above institution. She was trained at the Albert Edward Infirmary, Wigan, where she attained the position of assistant matron. She was then made night superintendent of the Victoria Hospital, Burnley.

Tunbridge Wells Eye and Ear Hospital.—Miss Edith Jones has been appointed Matron of the above hospital. She was trained at the Devizes Hospital and afterwards worked at the Royal Ophthalmic Hospital, Moorfields.

### Post-Graduate Clinics for Nurses.

By a Trained Nurse.

### XXIII.—A TYPICAL REST-CURE.

Many nurses break down hopelessly after a few months of "rest" nursing, and become themselves victims to a disease they have learnt most to dread. And I must confess that some of the most trying rest patients I have ever met have belonged to the nursing ranks, and they have exhibited the whole range of irritable, trying ways which they in health had so deplored in their patients. While Matron of a rest hospital it was by no means an uncommon incident for me to receive during the middle of the night a hasty and peremptory summons through the night nurse. "Would I come at once to patient A——, who desired most particularly to see me." Rousing out of a comfortable sleep after a hard day's work I sympathetically hastened to the bedside of the sufferer, who would greet me after some such fashion as this: "Oh, Matron, I felt so sleepless and lonesome I wanted a nice cheery chat with you. Do sit down for half an hour and have a nice talk. I'm so nervous and homesick among all these strangers." Now this was quite unconscious selfishness. Such a patient quite forgot—so absorbed would she be in her own symptoms and condition—that while she might possibly make up next day for the broken night, to which she was probably accustomed, to me that lost sleep would never be regained. The hospital breakfast bell would ring with a punctuality painful enough to me after so disturbing an influence in the night's harmony, while, if she happened to be asleep her breakfast would be kept hot until such time as she took it into her head to awake. Or she could take sundry siestas during the day, while poor matron would be vainly trying to conceal from staff and callers the tired yawns with which she listened to "ward reports" and long conversations. In dealing with the work of a nurse in connection with a Weir-Mitchell case, it will, perhaps, be more practical to describe a typical normal case and the routine nursing thereof.

The first general proposition to lay down so far as the "rest" nurse is concerned is that no nurse should combine massage with the care of her patient. All the leading nerve-doctors in the United States affirm that the strain of caring for a rest patient is quite enough without adding to this the exhaustion of massing. And the separation of the office of masseuse from that of nurse is necessary also in the interests of the patient. "A tired nurse is a bad masseuse; and sometimes she seems to take the patient's strength instead of giving it" is the axiom of a celebrated nerve specialist. Consequently he never allows the nurse on a nervous case to give massage, and in addition he gives his patients the very best conditions making for cure by strictly limiting the number of cases his masseuse may undertake each day. "No woman—even though she have the constitution of an Hercules—should give massage to more than five or six persons daily, and this number will be impossible unless she take each day two quarts of milk, in addition to a very generous diet, and lies down to rest between cases," he says. And his views have authority enough to find no questioners. A tired, irritable masseuse has a very bad effect on nervous patients, who are mostly very susceptible to the influence of "temperaments." I have known a physician change a masseuse—a perfectly skilful and competent woman from the professional point of view—just because the patient "did not like her." To receive massage or nursing from a person antipathetic or objectionable personally to the individual patient is held to be of very little value to a neurotic, whose likes and dislikes seem to act so powerfully for good or evil on his recovery. Taking a typical rest case, undergoing the treatment for the first time, the proscribed period for remaining in bed will usually be from six to eight weeks. Some very bad cases may be kept lying down for some two to three months, but such cases do not come under the normal rest-cure head.

Dr. Weir-Mitchell is very fond of dwelling in his addresses to students and young medical practitioners on the importance of "being quite sure when you order a patient to bed that you will be able to get her up again when you wish to do so." He himself had never experienced the difficulty, but many rest-cure doctors have found it an absolute impossibility to induce a patient put to bed to resume the ordinary normal way of living. I have myself seen women who had taken to their beds after being disappointed in life, or as the sequel to a domestic loss, and there they have remained for fifteen years, never once getting off the bed, although apparently quite well and strong. And with excellent appetitites and evident good constitutional condition their obstinate determination has been such that all the cleverest doctors have been quite unequal to the task of persuading them to leave their beds and walk.

After deciding to treat a rest-patient to a definite period of lying in bed—and this away from home—the next step is to choose a clever, sensible nurse, who can be relied on not only to nurse but to influence the patient, who has doubtless been picturesquely posing at home in the sympathetic atmosphere of frightened friends. The patient has declared she is too tired to read, her friends have read exciting novels for hours at a stretch; she has complained of the light—every curtain and blind has been drawn so that she has been living in semi-darkness, with lamp or gas burning day and night. She has imagined herself so susceptible to draughts that every chink and crevice in chimney, door, and window has been stuffed up with cotton-wool. Each varying whim and fancy as to diet has been gratified, the patient has lost self-control and will power, and is in a pitiable condition of hysteria.

The bed of the rest-cure patient should be a special care of the nurse, and should be most comfortable, even, and well-made. It should not rattle or creak, for this would annoy and irritate a neurasthenic patient whose fate it is for so long a time to find on that particular bed all the comfort and rest he will have. In some exceptionally mild cases the patient may have permission to be lifted for an hour each morning and night on to a couch "for a change." But this will be unusual, for the interest and excitement of the move will interfere with that complete and wholesome boredom, the production of which is the aim and object of doctor and nurse alike.

Looking over my note-book I find the complete record of a typical rest treatment case, Mrs. V., who by the way proved a most difficult person to manage. Her history was a normal one of "nerves" coming on for some two years, accompanied by a restlessness and sleeplessness which had reduced several members of the family to a condition bordering on her own. Mrs. V. was excitable and irritable. In addition to the condition of her nerves, she was suffering from atonic dyspepsia and chronic "liver." To relieve this she had been accustomed to indulge to an inordinate extent in hot-water drinking—a habit she had prescribed for herself.

### Presentations.

At the Ferèdale Infirmary, Sheffield, Miss Anne Pruett was presented by the nursing staff of the institution with a handsome chased silver teapot and other gifts on resigning her post as night superintendent to take that of superintendent nurse at the Sunderland Infirmary.

### The Power of Influence.

By a Matron.

To many one of the attractions of the nursing profession, especially as practised in hospital, is that it offers abundant scope for influencing others. "Enlarging our field of influence" are words that trip glibly off our tongues as we rise to a higher rank or enter upon a fresh sphere of work, and we start anew with fervid desires that others may profit by our example and experiences.

Influence is exercised in three ways—physically, morally, and spiritually. Some may be inclined to think that physical sway is of little consequence as compared with the other two. But it is not to be despised. Are we not sometimes profoundly influenced by an attraction that is perceptible to the senses? Not necessarily personal beauty or commanding stature, but an attractiveness which carries with it authority and demands attention. There are some whose word or look is all-sufficient; there are others who may imperiously command but fail to obtain obedience. Sometimes we come across those put in authority who have not the physical power of upholding the dignity of their office, whose time is spent in attempting to assert themselves, and in trying to ape the dignified manner which, unfortunately, does not come naturally, and we feel sorry for those people. Others, again, unconsciously exert an influence by an inherent force which cannot be explained, but which is sensibly conveyed by this power of physical attraction.

Whilst this particular form of influence is not given to all, the exercise of a moral influence is inevitable. It is a solemn responsibility which cannot be shirked, and a power which perhaps nurses wield to a larger extent than most people. We exercise it for good by the morality of our own lives, by the carrying out of high principles, by our strict sense of duty and honour, by example and by precept. A ward sister has specially abundant opportunities for putting this power in practice. Patients, probationers, nurses all look up to her as their model, her influence will be spread over her ward, and hers will be the accepted standard of professional integrity and virtue. The sphere may be wide or it may be limited, but there are none so insignificant as to be altogether outside the pale, and it is a truism to add that each of us must leave the world better or worse from having lived in it.

The power of spiritual influence as distinguished from moral influence is so subtle that to attempt to analyse it minutely would entail a higher flight into the region of ethics than the occasion warrants. Suffice it to say that hospital life may have brought some of us in contact with a kindred spirit whose thoughts and aspirations have blended with our own unrestrained by any barrier of reserve. It has been one of the greatest joys of our life, and we have found it to be the strongest human influence which could have been brought to bear upon our being.

Let us remember our influence is not limited to our own immediate circle; it is carried wherever sick people and nurses are to be found, and many are the beacon lights whose warnings we remember even though they have ceased to shine upon our shores—women whose work has carried them away to other parts of the circuit of action and influence, or who, having finished their nursing career, rest from their labours, and whose works do follow them.

### Victoria Commemoration Club for Nurses.

The secretary of the above club desires us to state that the nurses and others eligible are invited to join for the last six months of the year for a subscription of £1 1s., representing half the entrance fee, and half a year's subscription.

### Infant Incubators.

A company has been formed to introduce to the English public that form of infant incubator used by the French State. Opportunity has been taken of the Victorian Era Exhibition to show the apparatus at work and the babies in the process of being "hatched."

The origin of the invention is comical. M. Odile Martin, at the Jardin d'Acclimatation, had constructed a similar thing for rearing poultry, and Dr. Tarnier, when he saw it, deemed it an advance upon those used for infants, and requested the inventor to make one suitable for babies. This was done, and the "couveuse," as it was called, was introduced into the Paris Maternity Hospital in 1880. The present exhibit is their improved descendant. It is constructed of bright metal with glass in the side and in the doors. On the left side is a four-inch pipe, through which fresh air is admitted. Cotton-wool pads moisten and filter the air automatically before it enters the incubators. At the right-hand side a small metal boiler, heated either by a gas jet, a lamp, or electricity, keeps a constant flow of hot water, automatically maintained at a uniform temperature, circulating through a coil of piping which lies at the bottom of the incubator above the influx of air. Above both is a round disperser, and at the top of the incubator is a ventilating shaft. Thus the necessary supply of fresh air is admitted and the used air expelled. The self-acting valve by which the heat is regulated is well known; it is supplied to some gas metres and to other incubators. That exhibited last year at the Nurses' Exhibition in St. Martin's Hall had one on a similar principle. When the heat rises above the set point it lifts a cap, which in turn drops a slide controlling the space allowed for the inflow of hot water, and, consequently, the temperature inside the incubator is lowered, and *vice versâ* when the heat falls below the desired point the cap falls, the valves open wider, and more hot water runs into the heating coil.

A wire-woven hammock is hooked across, upon this rests a mattrass, and the baby (wrapped up) is deposited on the mattrass, when the doors are shut. The child is fed every two hours during the day, and every three at night.

The benefit of incubators—for there are many kinds—is questionable. They undoubtedly guard the prematurely born from many risks attending their peculiarly sensitive condition, and give the weaker a chance of life. Nevertheless, the question whether life is in itself a desirable boon under all circumstances has still to be answered, and the responsibility of bringing into the world and of keeping alive the unfit is gaining a wider recognition. A larger number of diseased persons and the shutting of many spirits in prison houses of defective flesh are the penalties that accompany some of the beneficent (?) scientific discoveries of recent years.

### A Generous Invitation.

The Earl and Countess of Jersey have generously invited about 600 boys and girls of the National Refuges for Homeless and Destitute Children, and the sailor boys of the "Arethusa" and "Chichester" Training Ships (Greenhithe, Kent), to spend the day at his lordship's beautiful seat, Osterley Park, Isleworth, on the 21st inst. The invitation has been gratefully accepted, and each of the homes and ships will be accompanied by their efficient brass band. Two hundred girls from the Sudbury and Ealing Homes will share in the festivities, which are invariably well arranged by the noble Earl and Countess.

### Mediæval Practice in the Care of the Sick.

V.—EARLY HOSPITALS.

As early as 650 A.D. Bishop Laudry had founded the Hospital of the Hôtel Dieu in Paris, placing it under the care of nursing sisters. These "Hospitalières" were united during the Pontificate of Innocent IV., under the rule of Saint Augustine.

During the Crusades, "several hospitals which were served by the Sœurs Hospitalières," says a writer in the "Dublin Review" for 1855, "were expressly founded for the reception of the sick pilgrims and wounded soldiers returning from the East, and bringing with them strange and hitherto unknown forms of disease and suffering."

"Some of the largest hospitals of France and the Netherlands originated in this purpose, and were all served by the Hospitalières." The Hôtel Dieu and La Pitié remained, indeed, under the charge of the same religious order until the nineteenth century. But the Sœurs Hospitalières were apparently the only regular order of nurses, until that enlightened thirteenth century, "fruitful in all those results which a combination of widespread suffering and religious ferment naturally produces, saw the rise of another community of compassionate women destined to exercise a far wider influence." These were the "Grey Sisters" or Franciscans. While the cloistered daughters of Saint Francis, known as the Poor Clares, served God by means of prayer and bodily austerities practised within the walls of their convents, the Friars, both Franciscan, Dominican, and others, organised "third orders" of women, not cloistered (not always even living in community, though they sometimes did), who were to devote themselves to active works of mercy. These women were not necessarily obliged to take vows of celibacy, but they were bound by certain rules and regulations, and united in visiting the sick, either in hospitals or at their own homes, and in the performance of other kindred works of mercy. All classes of society recruited the third orders, queens, princesses, ladies of rank, wives and daughters of burghers, poor widows, maidens, and occasionally also married women. It will easily be understood that some of these good people would exhibit a greater aptitude for nursing than others, and consequently the care of the sick came gradually to be regarded as a vocation apart, and arrangements were made to enable those who desired it to have such especial training as constant attendance upon sick persons could afford, and doubtless, also some kind of theoretical teaching.

The tourist in the Low Countries has probably seen or heard of the Beguines; it is said that these nuns acted as hospital sisters as early as the ninth century, and they were certainly settled in Liege and elsewhere about 1173. The Beguines wore conventional dress (or uniform), but they were free to leave their Order when they chose, and they had hospitals of their own, with a regularly appointed staff of medical men.

In 1443 a few of the Liege Beguines were summoned to Beaune to take charge of a large hospital founded by a chancellor of Philip the Good. This small nucleus of nurses were joined by other ladies from neighbouring districts, whom they trained, and at length expanded into an ordered called "Sœurs de Saint Martha." In England we know there were many hospitals for the benefit of the sick poor; Saint Bartholomew's, founded by Rahere in Henry I.'s reign, forms an interesting link between the Greek disciples of Æsculapius and modern science; and the poet Gower, in his will, has given us a peep into life at Saint Thomas' Hospital in the fourteenth century. He left 40s. to the master, 3s. 4d. to each sister, 20d. to each nurse, and 12d. to each patient, asking in return prayers for his departed soul. Gower also bequeathed 10s. to every leper house in London.

Leprosy was such a prevalent disease in the middle ages that Matthew Paris tells us 9,000 hospitals for patients suffering from it existed in Europe in his day. Dugdale's "Monasticon" contains a long list of such institutions in England, besides those which existed for the cure of other diseases. For instance, Saint Leonard's Hospital, York, re-founded by King Stephen, was for the reception of the poor and lame; it was nursed by eight sisters. In 1190 the Abbot of St. Albans founded a leper hospital for women on the same lines as a previously existing one near that town for men; it was served by Benedictine nuns; and, to give one more instance out of hundreds, there was a leper hospital outside the walls of Nottingham, to which, in 1226, Henry III. made a grant of "dead wood" for firing.

One feels that nurses for these lepers and "poor infirm" persons must have held much the same position as nurses in a "home for incurables" to-day. There is more promise of life in the words which tell us that St. Bartholomew's Hospital was to employ master, brethren, and sisters "for the entertainment of poor, diseased persons till they got well; of distressed woman with child, till they were delivered and able to go abroad; and for the maintenance (till the age of seven years) of all such children whose mothers died in the house."

### Minor Appointments.

City of Newcastle Union Infirmary.—Miss Beatrice Austin was appointed Superintendent Nurse of the above institution on July 16th. She was trained at the Jenny Lind Infirmary, Norwich. She took her certificate at the London Hospital, after working at the East London Hospital for Children, and remained on the private staff for a year. For the last four years she has been sister in charge of the male surgical and accident wards of the General Infirmary, Bedford.

Brighton and Hove Hospital for Women (Northern Branch).—Miss Margaret C. Lancaster, L.O.S., was appointed Sub-Matron of the above hospital on July 9th. She was trained at St. Thomas' Hospital, and has recently held the position of sister of the Birmingham Infirmary.

Joint Hospital, Kilwinning.—Miss Sara McGarrock has just been appointed Charge Nurse of the above-named institution. She was trained at the City of Glasgow Fever Hospital.

Ross Cottage Hospital.—On July 13th Miss Katherine Drury was appointed Assistant Nurse of the above institution. Miss Drury was trained at St. Mary's Infirmary, London, where she remained some years.

Birmingham and Midland Eye Hospital.—Miss Evelyn Hilton was appointed Assistant Matron at this institution on July 15th. She was trained at Newark Hospital, and has held an appointment at Charing Cross Hospital.

Sunderland Union Infirmary.—Miss A. Pruett has been appointed Superintendent Nurse at the above institution. She was previously night superintendent at the Fir Vale Infirmary, Sheffield.

Guildford Union Infirmary.—Miss Clarissa Cooper has been appointed Night Nurse at this infirmary. She has been working at the Claybury Asylum.

### Wants and Workers.

Can any of the readers of The Hospital tell me where I can get some letters from subscribers to the Surgical Aid Society, Salisbury Square, for an invalid who has been bed-ridden over thirty years. The letters are wanted for a water bed. I shall be thankful for any help.—G. M. Warren, Colville House, Wellesley Road, Colchester.

Wanted, a home where a lad of 16, who has been flat on his back for seven years (the result of spinal meningitis) could be received. He can now use his arms and move his legs, and the doctor thinks recovery is only a matter of time. Sea or country air would be very beneficial. His father is poor, but a small sum could be paid for his maintenance. Electricity and massage would be very desirable if possible. Address Mrs. Lee Williams, Lower Wick House, Worcestershire.

### Novelties for Nurses.

Messrs. R. and W. Wilson and Sons (Limited), of 90, Wardour Street, and 75, Dean Street, are to be congratulated on the very useful and comprehensive catalogue they have just issued. To hospitals and other institutions about to furnish it will be found invaluable, a glance at the contents enabling the purchaser to arrive at a decision as to the nature and quality of the various articles he may require with an approximate idea of the outlay it will necessitate. Everyone recognises the hopelessness and sacrifice of both time and patience of entering a large establishment to select a certain number of articles without having worked out the details quietly beforehand. With so complete a catalogue as Messrs. Wilson's, however, in hand the purchaser finds his way made comparatively easy. The tin and Japan ware are worth special attention, the enormous variety in baths alone deserving a visit. There are hip baths, sitz baths, Turkish (portable) baths, reclining baths, and a most fascinating plunge bath, with spray, douche, wave and shower accessories. A new upright needle spray bath will be found a boon to those who cannot afford the expense of a fashionable watering place. Gas and self-heating baths are another novelty invaluable for the colonies and country houses, where it is unusual to find a bath-room with hot water laid on. The hospital bath for bedside use is one we can particularly recommend for simplicity, good workmanship, and the ease with which it can be conveyed from one place to another. The slipper bath is another excellent description of bath for an invalid, as from its construction, after the patient is inside, it is easily closed up and the steam prevented from escaping. Leg baths and bed baths will be found very convenient under appropriate conditions, and are well worth inspection. There is also a wide range of cooking utensils of every size and shape, and the devices to save labour and ensure perfection are as numerous as they are ingenious. Warren's Invalid Cooker is an admirable invention. It is on the principle of a Bain Marie, which is unquestionably the best for cooking anything in which the strength and flavour is to be maintained. Beef tea made in this way is especially nourishing and good. Then, in addition to these, there are strainers and sausage cookers, vegetable warmers, and stock pots that would delight the most fastidious housewife. Tea urns for ward use are excellent value for the money, and are good in shape, and capable of containing from one to twelve gallons. The cabinet folding washstand is a capital contrivance for a bed-sitting room, where it would form quite an ornamental piece of furniture when not in use. Indeed, nothing that can add to the comfort and luxury of modern life has been overlooked, and in conclusion we should advise our readers to go and see for themselves. Under any circumstances a visit to Messrs. R. and W. Wilson's establishment would be a liberal education, while from the number and variety of the latest appliances that are now on view it could not fail to be interesting.

### REFRESHING PERFUMES.

There can be no two opinions as to the refreshing qualities of good eau de Cologne. It is especially valuable in the sick room. A few drops added to the water form the most cooling ablution in the hot weather, or, sprayed into the air, it is often most acceptable to the invalid. Care should, however, be taken that none but the best perfumes are used, as inferior kinds cannot fail to be distasteful, especially in sickness. There is no better eau de Cologne than that well known as 4711, made by Messrs. Mühlens, and to be purchased at 62, New Bond Street. To those to whom the perfume of violets is acceptable Messrs. Mühlen's Rhine violets can be recommended as a most charming scent, possessing the true aroma of the violet.

### CHARTS FOR NURSES.

Miss C. M. Löhr, member of the Royal British Nurses' Association, has just designed a useful little book for nurses. It contains twenty-two charts for day nurses' report, and the same number for night nurses' report on the other side of the sheet. Blank spaces are left for date, time, nourishment, quantity ounce, stimulant ounce, medicine ounce, sleep, hours and minutes, extras, and remarks. At the foot of page the totals of all find space, with temperature, pulse, respiration, bowels, and urine notes. When full the chart can be torn out by the perforations at the side. Messrs. Grellier and Sons, 211, Gray's Inn Road, are the publishers.

### Our American Letter.

Practical steps are being taken by the Superintendents' Convention to ascertain the curriculum given in the nurses' training schools in the United States with a view to obtaining a uniform standard of instruction.

Fifty-eight out of sixty superintendents invited to furnish statistics replied, with the result that much valuable information has been collected. In the majority of schools the two years' course of instruction, with a percentage of marks varying from 50 to 90 per cent., and a good report for conduct and work is necessary in order to obtain a diploma, but the three years' course is growing in favour, and nearly half the schools attached to the larger hospitals have adopted it.

There is a great difference in the date of entry, some institutions filling up vacancies as they occur, whilst others only admit students at the beginning of a term, which is once, twice, or thrice a year as the case may be.

The one thing that trends towards uniformity is the nearly universal use either of Miss Isabel Hampton's or Miss Clara Weeks' text book on nursing. Vacations are arranged in some institutions according to convenience, but a large proportion give holidays during the summer time.

American women are taking up the profession of dentists with something like enthusiasm. Seven of the 130 students at the North-Western University Dental School, Chicago, were women; and Miss Alice M. Steeres, a certificated nurse, trained at the Boston Training School for Nurses, took her degree D.D.S. after a three years' course of study. Last year Miss Steeres obtained 100 per cent. of the marks in her examination, and the previous year 95 per cent. The Grand Opera House, Chicago, was well filled with an enthusiastic assembly on the occasion of Miss Steeres taking her degree, and Professor Gilmer presented her with a case of instruments on behalf of himself and the class. Dr. Steeres has begun practice in the city.

Money has been given to erect a new operating-room of the Elliot Hospital Training School for Nurses. This hospital, the gift of Mrs. Mary Elizabeth Elliot, which stands on a hill overlooking the city of Manchester, New Hampshire, U.S.A., contains thirty beds, and was opened in 1890. Five years later the Louisa M. French emergency ward, in the heart of the city, was opened as an adjunct, and the latest addition is the nursing home situated close by the hospital proper. Miss Mary Elizabeth Barr is the matron, and she is assisted by a head nurse and twelve student nurses. Two rooms in the home are devoted to maternity cases. It seems a comfortable and well-managed training school, but the number of beds is too small to give much experience.

Dr. P. A. O. Mallison read to the nurses at the regular meeting of the N.Y.C.T.S. Alumnæ Association an interesting and much-needed paper on narcotics, in which he exposed a number of quack tonics and "cure-alls." The almost universal use of sedatives is exercising a baleful effect on the American people, quite young children being martyrs to "nerves."

### Everybody's Opinion.

[Correspondence on all subjects is invited, but we cannot in any way be responsible for the opinions expressed by our correspondents. No communication can be entertained if the name and address of the correspondent is not given, or unless one side of the paper only is written on.]

### NATIONAL HOSPITAL (ALBANY MEMORIAL).

MR. B. BURFORD RAWLINGS writes: The little piece of criticism which finds a place in your notice of our arrangements for "Queen's Day" is entirely fair and kindly. If only in justice, however, to the celebrated florists who helped us, may I say that the choice of Iceland poppies and cornflowers respectively for two of the wards was due to a wish expressed by the sisters? Iceland poppies are never large, they are the smallest of all; but their true colours harmonise so perfectly that they are favourites for decorative purposes, and usually prove more enduring than the larger sorts. In our case weather must have affected them, and many shed their petals before the day was over. About the second week in July, as practical gardeners know, there comes a pause in the procession of hardy flowers. The red lilies, foxgloves, iris, and delphiniums have gone out of blossom, and not for another ten days or a fortnight can we look for the gladiolas, sunflowers, dahlias, or hollyhocks. These eight groups comprise perhaps the grandest blooms of all, and not one of them was available on our gala day.

### NARROW-MINDED NURSES.

"ONE WHO DESIRES BREADTH" writes: I should be obliged if you will kindly find room in your columns for a few remarks *apropos* of "Narrow-minded Nurses." I daresay there is a good deal of truth in what "Onlooker" says, but surely there is no need to encourage us in our narrow-mindedness. During the first year or so in hospital I know from experience that a nurse has no time to think of anything beyond the hospital walls—her whole heart must be given to her work; but later on, when the worst of the routine is learnt and she has in great measure got over the constant strain on the mind, then, I think, is the time for her to beware of becoming that uninteresting thing, "a woman of one idea." There is no need to "live two lives," only to broaden one's views and sympathies enough to remember that there are many other hard workers in the world, and to take an intelligent interest in their work, as they do in ours. What would one think of the clergyman who was so full of his services, clubs, and guilds that he could not even listen with any sympathy to the story of his people's joys or sorrows, or to an account of the work of the hospital or district nurse? And yet this very same selfishness (for that is what it comes to) is what we are guilty of when we can talk or take an interest in nothing beyond our work or our "cases." We should not be so much taken up with our "work of lowly love" as to forget the cultivation of the "fellowship of hearts."

### THE NURSING SYSTEM OF THE CITY OF LONDON INFIRMARY.

"TRUTH" writes: In the "Nursing Mirror" of July 3rd there is an article on "Untrained Nurses in Workhouse Infirmaries," specially referring to the City of London Union Infirmary. As the statements there made are likely to create quite a wrong impression on the public mind, I venture to give a few facts. The infirmary in question is divided into nine landings. On each of these there is either a trained hospital nurse in charge or a nurse who has had many years' experience in the infirmary, and under her there are two probationers. As for the epileptics, the ward occupied by them forms part of one of these landings, for which the charge nurse is responsible. She, however, is allowed an extra probationer, so that the epileptic ward is never without a nurse by night or day, the nurse who was allowed to leave being a temporary nurse at £2 2s. per week, employed because of a vacancy on the staff, now filled. Naturally there must be a larger percentage of untrained women than trained, unless the infirmary be divided on a different system and more than one trained nurse be responsible for a landing, thus allowing for fewer probationers, but this is quite unnecessary, considering the class of cases admitted to this infirmary. The night duty is performed by some permanent night nurses under the old system and some advanced probationers on alternate day and night duty, all being under the charge of a trained and experienced night superintendent. The City of London Infirmary never was nursed as it is at the present time, and to prove the care bestowed on the patients by the nurses a visit to the wards only is necessary.

### THE HONITON DISTRICT NURSING ASSOCIATION.

DR. SHORTRIDGE writes: With respect to a paragraph which appeared in your last issue *re* the above, I think it calls for some comment. I will not dwell upon the want of frankness of the person who inspired it beyond saying that any nursing association that looks for a doctor's co-operation in his practice ought to consider in every respect his wishes with regard to its formation and management, especially in so far as it may affect his private or professional interests. During a period of over 20 years in which I have been engaged in practice, I have seen much of nursing and nurses—both personally and professionally. I owe them much, and many of your nursing readers who have worked under my directions have, I am sure, never failed to find me both courteous, gratefully appreciative, and desirous of furthering their welfare and interests in every way. I may be forgiven for thinking that I am perfectly as well able to judge of the nursing needs of my practice as any lay body, no matter how admirably constituted. I disagree entirely with the rules and management of the Honiton District Nursing Association, and it is not my intention to permit any interference by its nurses in my practice, nor can I admit for a moment its right to pauperise my private patients by its eleemosynary assistance. In the face of much that has happened, professional boycotting and social avoidance, any private patient of mine asking me to work in conjunction with the nurses of the District Nursing Association (and against them there is no personal grievance) would be guilty of such an amount of bad taste as to make a withdrawal from the case both easy and necessary. As it is obvious that many of our patients and sympathisers were desirous of seeing a district nurse in association with us in our poorer practice, and as this desire was accompanied by substantial offers of assistance, we have preferred to appoint an independent one who will work under our immediate orders and supervision, untrammelled by rules and regulations that we consider superfluous. One who will best carry out our ideas of what is most beneficial to the patients who may come under their charge. I am happy to say that the responses to our advertisement have been both numerous and cordial, and the chosen applicant will have no reason to complain either of the personal treatment accorded to her by the Honiton District medical men or their appreciation of her efforts in the way of duty.

### Notes and Queries.

The contents of the Editor's Letter-box have now reached such unwieldy proportions that it has become necessary to establish a hard and fast rule regarding Answers to Correspondents. In future, all questions requiring replies will continue to be answered in this column without any fee. If an answer is required by letter, a fee of half-a-crown must be enclosed with the note containing the enquiry. We are always pleased to help our numerous correspondents to the fullest extent, and we can trust them to sympathise in the overwhelming amount of writing which makes the new rules a necessity. Every communication must be accompanied by the writer's name and address, otherwise it will receive no attention.

*Lady Sanitary Inspectors.*

(323) Can you tell me whether it is correct that the examination fee for sanitary inspectors is £4 4s.? Also whether it is the case that there are already many women qualified, but practically no posts available?—*A. E. Law.*

As regards fees, write direct to the Secretary, Sanitary Institute, 72 and 74, St. Margaret's Street, Regent Street, W. (2) There is great difficulty in ladies obtaining employment.

*Old Soldiers' Charity.*

(324) Can you kindly tell me if there is any home for old soldiers, or those with incurable diseases, where they will receive care and attention on paying a small amount?—*Florence Burnell* (Matron).

See pp. 906 and following of "Burdett's Hospitals and Charities, 1897," for Institutions for Incurables. The Royal Commission of the Patriotic Fund, 53, Charing Cross, S.W., administer various funds for soldiers a their families.

